# Exogenously Applied Triacontanol Mitigates Cadmium Toxicity in *Vigna radiata* L. by Optimizing Growth, Nutritional Orchestration, and Metal Accumulation

**DOI:** 10.3390/toxics12120911

**Published:** 2024-12-14

**Authors:** Saba Mudassar, Shakil Ahmed, Rehana Sardar, Nasim Ahmad Yasin, Muhammad Jabbar, Maximilian Lackner

**Affiliations:** 1 Institute of Botany, University of the Punjab, Lahore 54590, Pakistan; 2 Department of Biological and Environmental Sciences, Emerson University, Multan 60000, Pakistan; 3 Faculty of Agricultural Sciences, University of the Punjab, Lahore 54590, Pakistan; 4 Faculty of Bio-Sciences, Cholistan University of Veterinary and Animal Sciences (CUVAS), Bahawalpur 63100, Pakistan; 5 Department of Industrial Engineering, University of Applied Sciences Technikum Wien, 17 Hoechstaedtplatz 6, 1200 Vienna, Austria

**Keywords:** cadmium, growth, mung bean, proline, TRIA, *Vigna radiata*

## Abstract

Cadmium (Cd) is one of the foremost phytotoxic elements. Its proportion in agricultural soil is increasing critically due to anthropogenic activities. Cd stress is a major crop production threat affecting food security globally. Triacontanol (TRIA) is a phytohormone that promotes growth, development, and metabolic processes in plants. The current study explicates the mitigation of Cd toxicity in *Vigna radiata* L. (mung bean) seedlings through the application of TRIA by a seed priming technique under Cd stress. The role of TRIA in improving metabolic processes to promote *Vigna radiata* (mung bean, green gram) vegetative growth and performance under both stressed and unstressed conditions was examined during this study. To accomplish this, three doses of TRIA (10, 20, and 30 µmol L^−1^) were used to pretreat *V. radiata* seeds before they were allowed to grow for 40 days in soil contaminated with 20 mg kg^−1^ Cd. Cd stress lowered seed germination, morphological growth, and biomass in *V. radiata* plants. The maximum root and shoot lengths, fresh and dry weights of roots, and shoot and seed germination rates were recorded for TRIA2 compared with those of TRIA1 and TRIA3 under Cd stress. In Cd-stressed *V. radiata* plants, TRIA2 increased the content of chlorophyll *a* (2.1-fold) and *b* (3.1-fold), carotenoid (4.3-fold), total chlorophyll (3.1-fold), and gas exchange attributes, such as the photosynthetic rate (2.9-fold), stomatal conductance (6.0-fold), and transpiration rate (3.5-fold), compared with those in plants treated with only Cd. TRIA seed priming increased nutrient uptake (K^1+^, Na^1+^, Mg^2+^, and Zn^2+^), total phenolic content, total soluble protein content, and DPPH (2,2-diphenyl-1-picrylhydrazyl) activity. Additionally, TRIA2 significantly reduced the quantity of Cd in the plants (3.0-fold) and increased the metal tolerance index (6.6-fold) in plants contrasted with those in the Cd-treated plants. However, TRIA2 promoted plant growth and biomass production by lowering Cd-induced stress through modifying the plant antioxidant machinery and reducing oxidative stress. The improved yield characteristics of *V. radiata* seedlings treated with TRIA suggest that exogenous TRIA may be used to increase plant tolerance to Cd stress.

## 1. Introduction

Potentially toxic elements poisoning soil is one of the foremost important abiotic stresses in the world [1]. Because of its nonbiodegradable and persistent characteristics in the ecosystem [2], metal contamination in soil is a serious concern. The main cause of increasing heavy metal pollution is human activity. The use of industrial effluents for irrigation methods has the potential to increase the content of heavy metals in soil, which can also affect the electrical properties, salinization, chloride content, total dissolved solids, pH, and levels of dissolved oxygen and phosphate [3]. Owing to the global freshwater shortage, irrigation by using wastewater containing potentially hazardous heavy metals is a common practice in some countries, although this is illegal [4]. Owing to Pakistan’s water deficit, the use of wastewater in agriculture will likely increase quickly [5]. Wastewater and floods result in sedimentation on residential and agricultural lands [6]. Floodplain soils and polluted sediments are damaging the environment, the safety of agricultural products, and, consequently, human and animal health [6]. In farmed areas, heavy metal pollution prevents plants from growing and lowers food quality [1].

Cadmium (Cd), along with other polluting metals, is frequent in arable areas worldwide due to natural weathering of rocks and agricultural and industrial practices resulting in agricultural yield loss [7]. Cd is listed as the seventh most hazardous element out of 20 [8] and is considered a main cause of concern because it is poisonous and mobile even at low concentrations [9]. The primary sources of Cd in the environment are agricultural sources, including pesticides and chemical fertilizers, as well as those associated with mining and industry [10]. The permitted limit for Cd^2+^ is 0.005–0.02 mg/L for plants and 1 mg/L for animals according to USEPA [11]. Cadmium harms plants physiologically, morphologically, molecularly, and biochemically [12]. Crops that receive wastewater irrigation are more likely to accumulate Cd, which inhibits seed germination, slows down root and shoot growth, decreases the plant height overall, reduces the number of leaves per plant, and, ultimately, kills plants [10]. Plants exposed to Cd exhibit distorted chloroplasts, reduced water and nutrient absorption and retention, and poor transpiration and photosynthesis [13]. Plants contain more reactive oxygen species (ROS), including H_2_O_2_, OH, and O_2_^1−^, under Cd stress, which also increases oxidative stress [14]. Cd can disrupt the photosynthetic system, resulting in reduced chlorophyll synthesis, and induce leaf chlorosis, necrosis, reduced plant growth, and other effects [11].

Priming enhances the physiochemical and metabolic activities required for appropriate seed germination. A higher germination percentage, improved radicle and plumule growth, uniform crop stand, and higher productivity are salient outcomes of seed priming [15]. Furthermore, seed priming increases nutrient uptake, activates enzymes, enhances plant stress resilience, and improves biomass production in the stressed environment [16,17,18].

Several endogenous and exogenous growth regulators enable plants to mitigate environmental stresses [19]. Triacontanol (TRIA, C_30_H_61_OH, CAS no. 595-50-0), a saturated primary alcohol that was initially discovered in alfalfa [20], is considered a novel plant growth regulator (PGR) that can modify numerous physio-biochemical phenomena in agricultural plants [21]. Plant species including *Medicago sativa*, *Oryza sativa*, and *Jatropha curcas*. naturally contain TRIA in their epicuticular wax. TRIA promotes plant development and increases plant growth when given exogenously to most crops at low concentrations [22]. When applied exogenously, it improves plant biomass, photosynthetic pigments, gas exchange parameters, mineral nutrient uptake, antioxidant enzyme activities, yield, and quality features [23]. Furthermore, TRIA alters the anatomy of leaf, stem, and vascular tissue systems in plants [24]. TRIA controls the expression of genes to suppress or amplify stress responses. The critical functions of TRIA in plant responses to abiotic stressors such as acid mist, cold, drought, heavy metals, and salt stress have been well characterized [25]. By increasing biomass production, chlorophyll content, gas exchange attributes, mineral nutrient uptake, and the antioxidant defense system, its application reduces the harmful effects of these stressors on plants [26].

*Vigna radiata* L. (mung bean) is the chief nutritious and economical grain legume belonging to the Leguminosae family. It offers basic nutritional protein benefits, making it a great choice for vegetarians. Despite its short lifespan, it contributes significantly to major agricultural systems and increases soil fertility. Although *V. radiata* can withstand different environmental conditions, its yield is still declining [27]. In addition to some regions of Africa and Australia, *V. radiata* crops are commonly grown in Asia. Currently, Asia produces 90% of the world’s *V. radiata*, with China, Pakistan, India, and Thailand being the continent’s top producers [28]. *V. radiata* is mostly cultivated as a summer crop in Pakistan; however, its average production is quite poor compared with other countries. Pakistan imports pulses in significant quantities to manage its rising protein demand [29]. Recent advancements in agriculture, molecular breeding, and integrated multiomics have promoted heavy metal resistance in many crop plants. However, these sophisticated methods are merely applicable in laboratory settings and are not very adaptable by farmers [30]. Moreover, there is a lack of research concerning the effects of seed priming with TRIA on the germination, growth, and maturity of *V. radiata* seeds under Cd stress. The present study aimed to investigate the impact of TRIA priming on seed germination, various physiochemical traits, and growth in Cd-exposed *V. radiata* plants.

## 2. Materials and Methods

The Roshan Seed Centre in Lahore provided certified *V. radiata* seeds of the Azri variety. *V. radiata* seeds were sterilized for 3–5 min by soaking them in 0.5% sodium hypochlorite solution and thoroughly rinsing them three times with distilled water. *V. radiata* seeds were primed for 8 h at room temperature with various concentrations of Triacontanol (TRIA) (10, 20, and 30 µM), referred to as TRIA1, TRIA2, and TRIA3, respectively. Following the priming process, the primed seeds were carefully washed thoroughly before being spread on blotting paper to air dry them at room temperature. As a control, seeds that had not been primed were used. Thirty-two clay pots were used for the experiments. With cadmium chloride (CdCl_2_), the soil (7.4 kg) in each of the allocated clay pots was contaminated with 20 mg kg^−1^ Cd. The soil that contained Cd spikes was subjected to Cd treatment. Both the Cd-contaminated and non-Cd-contaminated soils were placed in their designated pots. A randomized complete block design (RCBD) was used for this experiment to decrease systematic error, and after the pots had been filled, they were arranged according to their treatments. Additionally, four replicates of each treatment were set up. The pots were sorted and labeled based on their respective treatments and replication counts, and after 15 days, the soil was pretreated. Different levels of TRIA-primed seeds were sown in the appropriate pots.

### 2.1. Measurement of Growth Parameters

The harvest of plants was taken 40 days after sowing (DAS). Roots of plants were carefully taken out from the pots; they were cleaned properly. All the plants were subjected to measurements of several morphological parameters, including root length (cm), shoot length (cm), plant length (cm), leaf area (cm^2^), and number of leaves.

### 2.2. Biomass Production Assessment

*V. radiata* plants were harvested, and their fresh weights, such as shoot fresh weight (g), root fresh weight (g), and total plant fresh weight (g), were measured via an electrical balance (Sartorius GmbH, model 1216MP 6E, Goettingen, Germany). The plants were dried for 72 h at 70 °C in an oven (Wiseven, type WOF-105, Republic of Korea) to determine the dry weight, including root dry weight (g), shoot dry weight (g), and total plant dry weight (g).

### 2.3. Plant Photosynthetic Pigment Determination

Chlorophyll *a*, chlorophyll *b*, and total chlorophyll content were determined via the Arnon [31] method, whereas the concentration of carotenoids was determined via the Davis [32] method. Fresh *V. radiata* leaves were manually crushed with a mortar and pestle, extracted with 10 mL of an 80% acetone solution, and then kept at a low temperature. The mixture was subsequently centrifuged at 10,000 rpm for 5 min. On a spectrophotometer (Uv-1800 240V. CAT. No. 206-25400-38 SHIMZDZU Corporation, Kyoto, Japan), measurements were taken at 480 nm, 645 nm, and 663 nm to determine the optical density of the supernatant.

### 2.4. Assessment of Cd Accumulation

*V. radiata* shoots were cleaned with water and dried in a drying oven for two days at 65 °C (Wiseven, Model WOF-105, Korea) to determine the concentration of Cd by making acid-digested samples. The dried shoots were mashed with a mortar and pestle and passed through 60-screen mesh. A total of 1.5 mL of HClO_4_ (60%) and 5 mL of HNO_3_ (70%) were applied to a 0.5 g sample of plant shoots. The solution was heated continuously until the brown color disappeared. By adopting the procedure of Moseley and Jones [33], the solution was cooled and diluted (1:1) with approximately 5 mL of HCl (density of 1.18 g mL^−1^) in a 25 mL solution of water. An atomic absorption spectrophotometer (GBC XPLOR AA-Dual) was employed to quantify the Cd accumulation concentration factor [34]. The Al-Farraj et al. [35] technique was used to find the accumulation coefficient (*AC*).

(1) $$AC\;factor=\frac{Concentration\left(roots\right)}{Concentration\left(soil\right)}\;$$

The %MTI (metal tolerance index) was determined according to Balint et al. [36], as described below.

(2) $$\%MTI=\frac{Dry\;weight\;of\;treated\;plants}{Dry\;weight\;of\;untreated\;plants}\times 100$$

### 2.5. Estimation of Mineral Content

The mineral content from the digested samples of the *V. radiata* plants was analyzed. A flame photometer (Model 410, Corning) was used to evaluate Na^+^ and K^+^ content according to Sagner et al. [37], whereas an atomic absorption spectrophotometer (GBC XPLOR AA-Dual) was employed to quantify the concentrations of Zn^2+^ and Mg^2+^, as suggested by Chapman and Dale [34].

### 2.6. Assessment of Soluble Protein

For estimating total soluble protein, a 2 g plant sample was homogenized with 4 mL of 1 N phosphate buffer (34 g K_2_HPO_4_ in 2 mL of distilled water) with the help of an ice-chilled mortar and pestle. The mixture was centrifuged at 6000 rpm for 15 min. The homogenization of 0.8 mL of the supernatant was carried out with Folin solution (4 mL) for 15 min by keeping it at room temperature. Each sample received Folin reagent (0.5 mL), which was then shaken and left at room temperature for approximately 45 min. Using a UV-1800 Shimadzu spectrophotometer ((Shimadzu, Kyoto, Japan)), optical density measurements were made at 715 nm. The quantity of total soluble protein was estimated via a bovine serum albumin (BSA) standard curve.

### 2.7. Total Proline Content Determination

The proline concentration was determined using the protocol of Bates et al. [38]. A 0.25 g ice-chilled sample of plant leaves was extensively vortexed after adding 10 mL of 3% sulfosalicylic acid. The mixture underwent filtering. First, 2.8 g of ninhydrin, 48.16 mL of 85% phosphoric acid, 72.8 mL of acetic acid, and 2 mL of glacial acetic acid were added to 2 mL of filtrate to prepare a homogenous mixture. The homogenate was kept in a water bath at 100 °C (N.S Engineering concern XMTG-9000). After one hour, the solution was chilled to stop the process. Toluene (4 mL) was vortexed vigorously for 30 s in the mixture to draw off the upper toluene phase that contained the proline–ninhydrin chromophore complex. The mixture was incubated at 25 °C for approximately 0.5 h. In a Shimadzu UV-1800 spectrophotometer, the absorbance was measured at 520 nm and contrasted with the proline standard curve [38].

(3) $$Proline\;\left(µ\frac{mol}{g}FW\right)=\left[\frac{µg\;proline/mlx\;ml\;of\;toluene}{115.5}\right]/\left[\frac{g\;of\;sample}{10}\right]\;\;FW\;=\;fresh\;weight\;(g)$$

### 2.8. Analyzing the Gas Exchange Parameters

The transpiration rate (*E*), net photosynthetic rate (*A*), and stomatal conductance (*gs*) were determined via the IRGA (infrared gas analyzer) LCA-4 system of Analytical Development Co. Ltd. (ADC, Ltd. 12 Spurling works, Pindar Road, Hoddesdon, Herts, UK). Between 10:30 and 11:30 am, the highest completely extended leaf was used to take readings for several parameters.

The activity of 2,2-diphenyl-1-picrylhydrazyl (DPPH) was measured for the scavenging of free radicals. In total, 1 g of *V. radiata* sample was obtained, and 10 mL of methanol was used to create a methanolic extract via the Chen et al. [39] technique. For this purpose, 5 mL of 0.1 mM methanolic extract and 1 mL of complete mixture were combined. Freshly made 2,2-diphenyl-1-picrylhydrazyl (DPPH) methanolic solution was stored in the dark. After one hour, the absorbance was observed at 517 nm through a Shimadzu UV-1800 spectrophotometer. In total, 1 mL of methanol was used to prepare the blank.

The percentage (%) of free radical scavenging activity was determined as follows.

(4) $$Activity\left(\%\right)=Scavenging\left[1-\left(\frac{A517nm,Sample}{A517nm,Blank}\right)\right]\times 100$$

A = Absorbance

### 2.9. Assessment of Total Phenolic Content

To estimate the total phenolic content, 2 g of fresh leaves and 10 mL of 80% aqueous methanol were heated at 65 °C for approximately 15 min. A total of 250 µL of Folin–Ciocalteu reagent (1 N), 5 mL of sterilized distilled water, and 1 mL of the extract were combined and then stored at 30 °C. To determine the total amount of phenols, the absorbance of the blue hue emerging in the reaction mixture was observed at 725 nm and compared to the standard curve of gallic acid [40,41].

### 2.10. Statistical Analysis

The gathered data were examined via one-way ANOVA and SPSS software (IBM-SPSS Statistics 20). Duncan’s multiple range test was used to segregate means for significant treatment at 0.05 *p*-value. The data represent the average of four replicate ± S.E. Additionally, principal component analysis (PCA) and correlation were performed via RStudio software 2023.09.1+494.

## 3. Results

### 3.1. Growth Parameters of V. radiata

Compared with the control plants, the plants whose seeds were primed with TRIA2 showed a 1.41-fold increase in shoot length. Compared with those of the Cd-only treated plants, the shoot length of the TIA2+Cd-treated plants increased 2.5-fold. The plant seeds treated with TRIA3+Cd also had a 2.3-fold greater length than those grown only in Cd-spiked soil without TRIA treatment, as shown in Table 1.

### 3.2. Assessment of Biomass

The plants subjected to Cd stress exhibited 0.6- and 0.4-fold lower fresh weights of roots and shoots, respectively, as compare to plants under control conditions. Reciprocally, the dry and fresh weights of *V. radiata* seeds increased, and Cd stress was alleviated in *V. radiata* seeds, as shown in Table 2. Moreover, TRIA2+Cd enhanced shoot and root fresh weight as well as total plant fresh weight by 5.7-, 3.8-, and 5.2-fold, respectively, through the amelioration of Cd stress in comparison with that in Cd-only treated plants. There was a slight reduction in the dry and fresh weights of roots and shoots in the case of TRIA3 compared with those in the case of TRIA2.

### 3.3. Estimation of Photosynthetic Pigments and Cadmium Uptake Content

The toxicity of cadmium negatively affects plant pigments in *V. radiata* (Table 3). The plants were harvested on 30 DAS. The plants whose seeds were primed with TRIA2 demonstrated the maximum content of chlorophyll *a*, which was 2.46-fold greater than the control plants, which were not subjected to any treatment. The plants treated with TRIA2+Cd also showed the greatest chlorophyll *a* content among those under Cd stress. Furthermore, TRIA diminished Cd stress and increased chlorophyll (*a*, *b)*, total chlorophyll, and carotenoid content in the TRIA1+Cd, TRIA2+Cd, and TRIA3+Cd groups. Compared with the control plants, the Cd-stressed plants exhibited decreases in chlorophyll *a* and *b* content of 1.6- and 1.76-fold, respectively, as depicted in Table 3.

The cadmium uptake content reached a maximum in *V. radiata* plants growing in Cd-susceptible soil, which presented 0.84 mg Cd g^−1^ DW. The seeds of the plants that were primed with TRIA alleviated the toxicity of Cd. The maximum alleviation was noted for TRIA2+Cd, which decreased the uptake of Cd by 66.67% compared with that in Cd-contaminated plants that were not treated with TRIA. The highest accumulation factor was calculated for the plants grown under Cd-spiked conditions, and it was alleviated by 20%, 66%, and 46.43%, respectively, in the seeds of the plants treated with TRIA1+Cd, TRIA2+Cd, and TRIA3+Cd. The metal tolerance index was higher in TRIA2+Cd plants than in Cd-only plants by 84.85% (Table 3).

### 3.4. Determination of Plant Nutrition

The uptake of plant nutrients, including Mg, Zn, K, and Na, is affected by Cd toxicity. However, the nutrient content recovered in control plants and plants under Cd stress primed with TRIA indicated that seed priming with TRIA2+Cd greatly enriched the nutrient uptake of Mg and Zn by 4.5- and 2.9-fold, respectively, compared to that in only the Cd-treated plants. The lowest nutrient concentration was noted in the plants affected with Cd toxicity, which were deprived of TRIA. Additionally, TRIA3+Cd increased Mg and Zn by 3.5- and 2.7-fold, respectively, compared with those in plants treated with only Cd (Table 4).

### 3.5. Assessment of Soluble Protein and Proline Content

**Figure 1:**
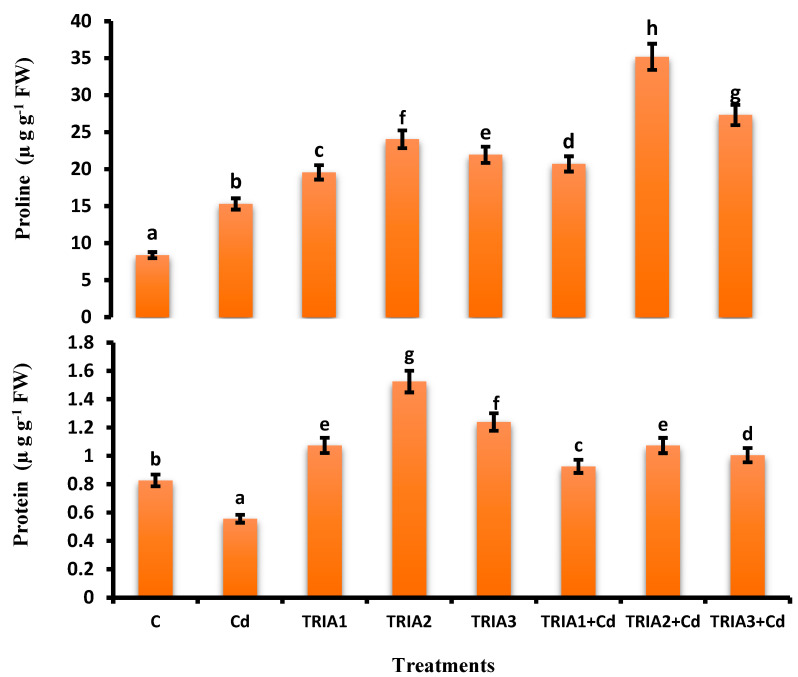
Effect of TRIA and Cd on proline and total soluble protein content of *V. radiata*. Values demonstrate means ± SE of four replicates (n = 4). Non-identical letters over error bars exhibit significant differences between the treatments at *p* ≤ 0.05. C = control, Cd = 20 mg kg^−1^ Cd, TRIA1 = 10 µM L^−1^ TRIA, TRIA2 = 20 µM L^−1^ TRIA, TRIA3 = 30 µM L^−1^ TRIA.

TRIA had positive effects on the soluble protein content of *V. radiata* plants. Compared with the plants under controlled conditions, the Cd-stressed plants presented a decrease in soluble protein content of 32.53%. However, the maximum soluble protein content was observed in the plants obtained from the TRIA2 seed priming. In contrast, the soluble protein content of the plants raised from TRIA seed priming and under Cd stress (TRIA2+Cd) was 20.74% lower than that of the TRIA2 plants (not under Cd stress), as shown in Figure 1.

### 3.6. Assessment of Proline Content

In comparison to the plants under controlled conditions, the proline synthesis in the Cd-affected *V. radiata* plants was significantly greater (82.57%). Compared with those of TRIA1, TRIA2+Cd, TRIA2+Cd, and TRIA3+Cd, which were not under Cd stress, the proline content of the plants that were under Cd stress from seeds primed with TRIA increased by 8.36%, 32.08%, and 32.25%, respectively. The plants supplemented with TRIA and not spiked with Cd showed lower proline content (Figure 1).

### 3.7. Estimation of the Photosynthetic Rate

**Figure 2:**
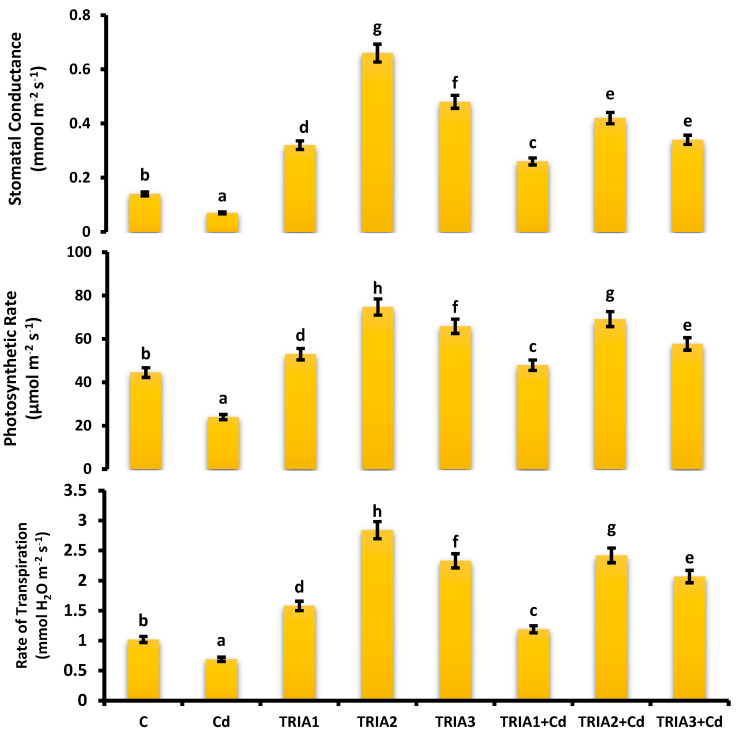
Effect of TRIA and Cd on photosynthetic rate, stomatal conductance, and transpiration rate of *V. radiata*. Values demonstrate means ± SE of four replicates (n = 4). Non-identical letters over error bars exhibit significant differences between the treatments at *p* ≤ 0.05. C = control, Cd = 20 mg kg^−1^ Cd, TRIA1 = 10 µM L^−1^ TRIA, TRIA2 = 20 µM L^−1^ TRIA, TRIA3 = 30 µM L^−1^ TRIA.

The pronounced effect of TRIA2+Cd on the photosynthetic rate was 2.8-fold greater than under only Cd conditions. However, the photosynthetic rate decreased by 1.08-fold in the TRIA2+Cd group compared with that in the TRIA2 group. The maximum photosynthetic rate was calculated for TRIA2, i.e., 74.89 µmol/m^3^/s, whereas the minimum photosynthetic rate was calculated for the plants under Cd stress, i.e., only 24 µmol/m^3^/s (Figure 2).

### 3.8. Determination of Stomatal Conductance

TRIA alleviated the toxicity of Cd stress and increased stomatal conductance in plants, i.e., TRIA1+Cd, TRIA2+Cd, and TRIA3+Cd, by 3.7-, 6.0-, and 4.9-fold, respectively, compared with that in only Cd-stressed plants. The highest stomatal conductance was recorded in the TRIA2 plants, i.e., 0.64 mmol m^−2^s^−1^, which was 4.17-fold greater than the control plants, as shown in Figure 2.

### 3.9. Estimation of the Transpiration Rate

Cadmium stress decreased the transpiration rate by 32% compared with the control. Compared with the control, seed priming with TRIA1, TRIA2, and TRIA3 increased the transpiration rate by 1.5-, 2.8-, and 2.3-fold, respectively. TRIA2+Cd increased the transpiration rate by 2.5-fold compared with that under only the Cd condition. The maximum transpiration rate was calculated in TRIA2, i.e., 2.81 mmol H_2_O m^−2^ s^−1^, and the minimum transpiration rate was recorded in Cd-affected plants, i.e., 0.69 mmol H_2_O m^−2^ s^−1^ (Figure 2).

### 3.10. Assessment of Total Phenolic Content

**Figure 3:**
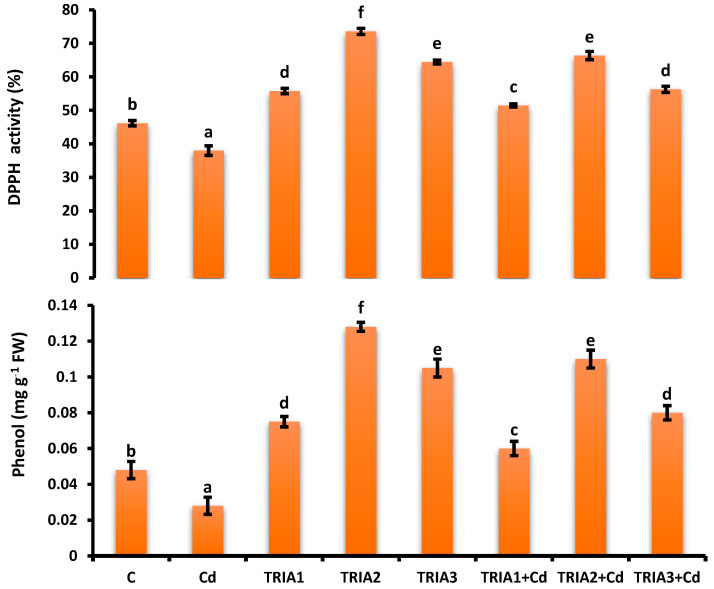
Effect of TRIA and Cd on DDPH and total phenolic content of *V radiata*. Values demonstrate means ± SE of four replicates (n = 4). Non-identical letters over error bars exhibit significant differences between the treatments at *p* ≤ 0.05. C = control, Cd = 20 mg kg^−1^ Cd, TRIA1 = 10 µM L^−1^ TRIA, TRIA2 = 20 µM L^−1^ TRIA, TRIA3 = 30 µM L^−1^ TRIA.

The total phenolic content was 41.67% lower in the Cd-affected plants than in the control plants. The maximum phenolic concentration was recorded in TRIA2, i.e., 0.13 mg g^−1^ FW, 2.7-fold greater than that of the control plants, i.e., 0.05 mg g^−1^ FW. The phenolic content in TRIA2+Cd-treated plants was 3.9-fold greater (Figure 3) compared with Cd-affected plants.

### 3.11. Estimation of 2,2-Diphenyl-1-Picrylhydrazyl (DDPH) Activity

The DDPH activity was 18% lower in the Cd-affected plants than in the control plants. The maximum DPPH activity was recorded for TRIA2, i.e., 72.12%, and for TRIA+Cd, i.e., 63.94%. In TRIA3 and TRIA3+Cd, the DPPH activity was slightly lower, i.e., 66.87% and 56.73%, respectively. Compared with that under Cd-only conditions, DPPH activity under Cd stress was increased by TRIA, as depicted in Figure 3.

### 3.12. Pearson Correlation

**Figure 4:**
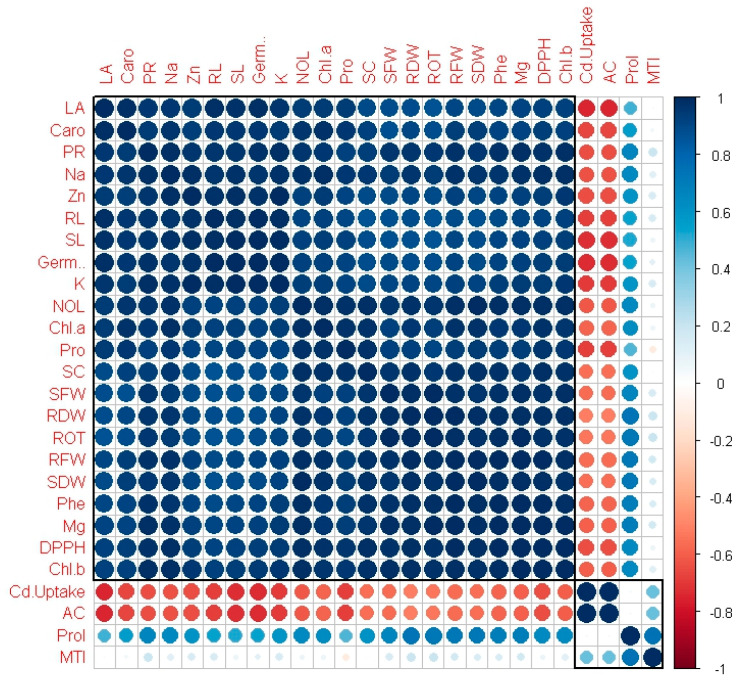
Pearson correlation for *V. radiata* under TRIA and Cd affect. Different abbreviated forms shown in figure as follows: Zn (Zn concentration in shoots), Mg (Mg concentration in shoots), k (K concentration in shoots), Na (Na concentration in shoots), Chl (chlorophyll), RL (root length), SL (shoot length), LA (leaf area), NP (net photosynthesis), SC (stomatal conductance), Caro (carotenoid content), Pro (protein content), Germ (germination percentage), MTI (metal tolerance index), AC (accumulation factor), Prol (proline concentration), Phe (phenolic level), NOL (number of leaves), SFW (shoot fresh weight), RFW (shoot fresh weight), RDW (root dry weight), SDW (shoot dry weight), ROT (rate of transpiration).

According to the Pearson correlation, there was a strong positive association between the Cd content and the AC factor. Significant negative correlations between the Cd concentration and characteristics such as the germination rate; shoot length; root length; chlorophyll *a*, chlorophyll *b*, total chlorophyll, and carotenoid content; total phenolic content; DPPH, soluble protein, and photosynthetic rates; transpiration rate; stomatal conductance; and Zn, Mg, K, and Na concentrations were detected. Figure 4 shows no positive or negative association between MTI and the other variables.

### 3.13. Principal Component Analysis

**Figure 5:**
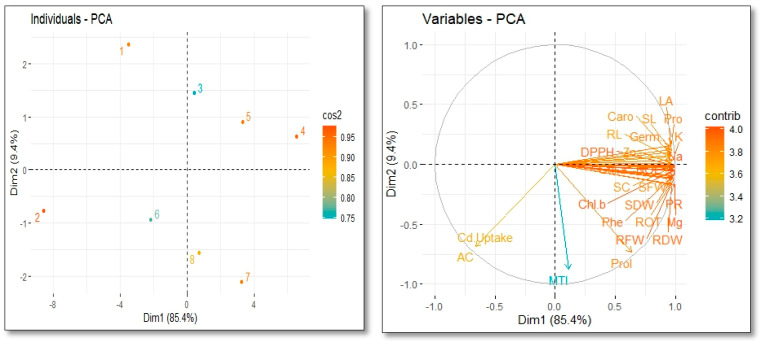
Loading plots of principal component analysis (PCA) demonstrated a relation between physiological parameters and growth under TRIA treatment and Cd on *Vigna radiate* L. Various abbreviations used in the figures are as follows: Zn (Zn amount in shoots), Chl (chlorophyll concentration), RL (length of root), SL (length of shoot), LA (leaf area), NP (net photosynthesis), TR (rate of transpiration), SC (stomatal conductance), Caro (carotenoid concentration), pro (protein), MTI (metal tolerance index), AC (accumulation factor), Prol (proline concentration), Phe (phenolic content), Ger (percentage of germination).

The relationships between the growth and physio-biochemical features of *V. radiata* grown in soil contaminated with Cd treated with TRIA were further demonstrated via the loading plots of the principal component analysis (PCA). More than 94.8% of the entire database consists of the first two primary components, Dim 1 and Dim 2, creating the largest portion of all the components. All the treatments were successfully dispersed across the entire database. Each contributes a percentage of the overall dataset, with Dim 1 contributing 85.4% and Dim 2 contributing 9.4%. The germination rate, root length, chlorophyll *a* content, chlorophyll *b* content, carotenoid content, total chlorophyll content, stomatal conductance, transpiration rate, photosynthetic rate, proline content, soluble protein content, DPPH content, total phenolic level, and Zn, Na, K, and Mg concentrations are positively related to the PCI. Figure 5 shows the negative correlation between the PCA and the amount of Cd in the shoot.

## 4. Discussion

Triacontanol (TRIA) seed priming increases seed vitality, which helps promote early seedling emergence [42] and increases the ability of plants to tolerate Cd toxicity for better establishment. TRIA priming helps to regulate and activate enzymes involved in stress tolerance and growth promotion in the early stages of embryogenesis, allowing for faster metabolism for germination. Our findings indicated dramatically enhanced growth in the presence of TRIA, which is consistent with the findings of Zaid et al. [23].

Across several plant species, heavy metal stress causes a decrease in root as well as shoot biomass production, which is generally regulated by plant hormones. The exogenously applied growth-promoting hormones mitigate heavy metal toxicity [43,44]. Our results also exhibited that the exogenous application of TRIA enhanced plant growth and biomass production under metal stress. In accordance with our findings, [25] also reported that seedlings treated with TRIA were found to have higher levels of CO_2_ and H_2_O absorption ability, as well as elevated activity of carbonic anhydrase. Naeem et al. [45] observed that exogenously applied TRIA augmented the biomass production and protein content in *Oryza sativa*. In the same way, Ahmad et al. [46] demonstrated the involvement of TRIA in the growth, stress alleviation, and yield of various crop plants.

Chlorophyll regulates the rate of photosynthesis, which adjusts physiochemical and metabolic activities related to plant growth and biomass production [47]. Our results exhibited decreased chlorophyll content and lower photosynthetic activity, resulting in poor growth in Cd-stressed *V. radiata* plants. Another researcher also found that Cd stress decreased chlorophyll *a*, chlorophyll *b*, and total chlorophyll [48]. Cd-stressed plants exhibit reduced activity of photosystem I (PSI) and photosystem II (PSII), in addition to the rate of the photosynthetic electron transport chain [49]. Cadmium ions hinder the absorption and translocation of Mg^2+^, leading to chlorophyll degradation [50]. Hayat et al. [51] observed reduced chlorophyll content in Cd-stressed plants, which confers our findings. Correspondingly, Handa et al. [52] noted a reduction in the photosynthetic content of metal-stressed plants. However, TRIA mitigates stress through adjusting stress tolerance processes. Islam and Mohammad [20] revealed that plants treated with exogenous TRIA showed modulated activity of enzymes, improved photosynthetic activity, and productivity under stress and normal circumstances. Nickel stress enhanced the biosynthesis of ROS (reactive oxygen species), augmented protein, DNA, and chlorophyll deprivation in maize plants [53]. Yet, plants treated with TRIA mitigated Ni stress and demonstrated higher enzymatic activity and improved photosynthetic activity and growth. The higher chlorophyll content in TRIA-treated plants during our study increased the rate of photosynthesis, resulting in improved root and shoot length as well as more biomass production. Perhaps TRIA augmented photosynthetic activity by increasing the activity of the Rubisco enzyme and upregulating genes involved in photosynthesis. TRIA improved the morphophysiological features and growth of canola and sunflower plants by increasing chlorophyll synthesis [54]. Similarly, our findings revealed that TRIA enhanced the chlorophyll *a*, chlorophyll *b*, total chlorophyll, and carotenoid content of *V. radiata* plants.

Phenolic compounds can chelate metals during heavy metal stress and directly absorb molecular species of active oxygen. Peroxidase oxidizes phenols, particularly flavonoids, and phenylpropanoids, then function in the phenolic system to scavenge H_2_O_2_. The chemical makeup of these compounds mostly accounts for their antioxidant effects. Plants can induce their phenolic metabolism in response to stressors (including heavy metal stress) [55]. To further understand how cadmium (Cd) and zinc (Zn) affect the metabolism of phenolic compounds and the mechanisms underlying heavy metal tolerance in *Kandelia obovata*, a pot experiment was carried out [56]. Perhaps the higher level of phenolic content synthesized during this study alleviated metal toxicity by chelating Cd and detoxifying ROS. This study suggests that TRIA-treated seedlings activated the PAL enzyme, which enhanced the synthesis of phenolic compounds. Zoufan et al. [57] noted that higher phenolic content improved the antioxidant system of plants to mitigate Cd stress. Yadav and Singh [58] also reported that the interactive effect of phenol-synthesizing hormones improved the antioxidative machinery of wheat under stress regimes. Equally, the augmented level of phenolics in TRIA-applied plants during this study mitigated Cd toxicity. In another study, Meza et al. [59] revealed that IAA affected DPPH scavenging by increasing the biosynthesis of phenolics plants. Correspondingly, the results of the current study confirm that growth-promoting TRIA enhanced DPPH activity in Cd-stressed plants to alleviate stress.

Ullah et al. [60] observed that a higher Cd concentration declines TI in chickpea cultivars. According to Menhas et al. [61], the improved antioxidative system of tolerant plant species correlates with stress mitigation and metal uptake potential under Cd stress circumstances. Higher MTI was observed in TRIA2-applied plants during this study. The reduced MTI level in plants growing in Cd-spiked conditions in absentia of TRIA treatment may be attributed to higher oxidative injury.

Metal contaminants negatively affect morphophysiological features of the stomata and other allied physio-metabolic procedures in plants. Mostly, metal toxicity declines stomatal conductivity [62]. The results of various studies demonstrate a reduction in stomatal conductivity through Cd stress in plants including pakchoi, mustard, marigolds, Holm oak, mastic shrub, *Bacopa monniera*, Populus, riparian *Salix variegata*, cucumber, cowpea, *Origanum vulgare*, *Ocimum basilicum*, and *Arundo donax* [63,64]. Yet, Fox et al. [65] reported higher stomatal conductivity in Cd-stressed corn, *Lactuca sativa*, mustard, and water hyacinth. TRIA seed priming increases plant growth and stress tolerance through increasing stomatal conductivity. In the same way, our results depicted that TRIA seed priming enhanced photosynthetic activity, the rate of transpiration, and stomatal conductance in developing plants in all treatments.

Proline, an amino acid, defends cellular integrity and maintains protein structure under a stressed environment by sustaining the water content, cellular turgidity, and osmotic potential of cells intricated in alleviating oxidative stress [66]. Plants subjected to stress exhibit higher proline content due to their innate potential to detoxify ROS [67,68]. Mayahi et al. [69] reported that TRIA heightened plant stress resilience by enhancing the biosynthesis of osmoprotectants including proline, total soluble proteins, and carbohydrates. Higher proline levels in Cd-stressed plants during the present study suggest the involvement of this osmoregulator in reducing metal-induced toxicity. Siddique and Dubey [69] revealed that proline content increased with the increasing concentration of CdCl_2_ treatment at 120 h of observation, while the least amount of proline was found in the control set. The increment in proline content from its normal level speaks to the presence of stress in the system. Proline participates in the osmotic adjustment of plant cells under stress has been reported to improve plant resistance to oxidative stress induced by heavy metal toxicity by scavenging ROS [68]. Likewise, our results revealed, in Tria-treated plants, that the relatively high level of proline relieved Cd stress.

## 5. Conclusions

In *V. radiata* plants, cadmium toxicity significantly reduced germination percentage, seedling growth, and biomass production by enhancing ROS levels. However, TRIA-treated seedlings exhibited an improved production of photosynthetic pigments, proline accumulation, nutrient uptake, and gas exchange attributes. The activity of antioxidant enzymes and the metal tolerance index were increased by TRIA, which reduced Cd toxicity. Our results support the use of TRIA2 (20 µmol L^−1^) to mitigate stress and preserve the growth of *V. radiata*. seedlings under Cd-contaminated conditions. However, further molecular analysis will reveal the complete mechanism by which TRIA reduces Cd stress and promotes development. Field experiments could be carried out to determine the efficacy of TRIA treatment to sustain crop yield under a contaminated environment.

## Figures and Tables

**Table 1:** Impact of TRIA on growth parameters and germination percentage of *V. radiata* under Cd stress.

Treatments	Growth Parameters					
	Shoot Length (cm)	Root Length (cm)	Total Length (cm)	Leaf Area (cm^2^)	No. of Leaves	Germination %
C	5.44 ± 0.10 ^b^	3.12 ± 0.11 ^b^	9.57 ± 0.24 ^b^	3.94 ± 0.21 ^b^	5.50 ± 0.46 ^b^	84 ± 4.00 ^b^
Cd	3.84 ± 0.15 ^a^	2.5 ± 0.09 ^a^	6.24 ± 0.25 ^a^	1.57 ± 0.10 ^a^	2.52 ± 0.40 ^a^	66 ± 3.00 ^a^
TRIA1	7.75 ±0.76 ^d^	4.65 ± 0.30 ^d^	12.4 ± 0.43 ^d^	5.93 ± 0.20 ^cd^	7.25 ± 0.27 ^d^	90 ± 2.00 ^bc^
TRIA2	9.11 ± 0.45 ^g^	5.76 ± 0.27 ^h^	14.87 ± 0.05 ^f^	7.98 ± 0.28 ^e^	10.5 ± 0.50 ^f^	100 ± 0.90 ^d^
TRIA3	8.54 ± 0.27 ^f^	5.01 ± 0.05 ^g^	13.55 ± 0.08 ^e^	6.12 ± 0.18 ^d^	8.25 ± 0.25 ^e^	95 ± 2.00 ^c^
TRIA1+Cd	6.92 ± 0.07 ^c^	4.33 ± 0.20 ^c^	11.25 ± 0.05 ^c^	5.01 ± 0.19 ^c^	6 ± 0.84 ^c^	85 ± 5.50 ^b^
TRIA2+Cd	8.12 ±0.75 ^e^	5.12 ± 0.06 ^f^	13.24 ± 0.27 ^e^	6.32 ± 0.34 ^d^	8.5 ± 0.40 ^e^	95 ± 3.00 ^cd^
TRIA3+Cd	7.45 ± 0.36 ^d^	4.87 ± 0.50 ^e^	12.32 ± 0.30 ^d^	5.47 ± 0.07 ^c^	7 ± 0.42 ^d^	90 ± 4.00 ^bc^

Values illustrate means ± SE of four replicates. Dissimilar letters on error bars showed significant differences between treatments at *p* ≤ 0.05. C = control, Cd = 20 mg kg^−1^ Cd, TRIA1 = 10 µM L^−1^ TRIA, TRIA2 = 20 µM L^−1^ TRIA, TRIA3 = 30 µM L^−1^ TRIA.

**Table 2:** Impact of TRIA on biomass assessment of *V. radiata* under Cd stress.

Treatments	Shoot Fresh Weight (g plant^−1^)	Root Fresh Weight (g plant^−1^)	Total Fresh Weight (g plant^−1^)	Shoot Dry Weight (g plant^−1^)	Root Dry Weight (g plant^−1^)	Total Dry Weight (g plant^−1^)
C (control)	0.94 ± 0.03 ^bc^	0.25 ± 0.02 ^b^	1.18 ± 0.02 ^b^	0.28 ± 0.02 ^b^	0.08 ± 0.02 ^b^	0.46 ± 0.01 ^b^
Cd	0.38 ± 0.01 ^a^	0.16 ± 0.03 ^a^	0.53 ± 0.03 ^a^	0.18 ± 0.04 ^a^	0.04 ± 0.04 ^a^	0.30 ± 0.05 ^a^
TRIA1	1.23 ± 0.09 ^c^	0.46 ± 0.09 ^d^	1.69 ± 0.14 ^d^	0.68 ± 0.01 ^d^	0.25 ± 0.05 ^d^	0.93 ± 0.32 ^d^
TRIA2	2.85 ± 0.10 ^f^	0.72 ± 0.06 ^g^	3.57 ± 0.06 ^g^	1.13 ± 0.26 ^g^	0.53 ± 0.08 ^g^	1.66 ± 0.04 ^h^
TRIA3	2.21 ± 0.12 ^e^	0.58 ± 0.05 ^e^	2.79 ± 0.16 ^f^	0.84 ± 0.09 ^e^	0.39 ± 0.04 ^ef^	1.23 ± 0.05 ^f^
TRIA1+Cd	1.01 ± 0.38 ^cd^	0.33 ± 0.09 ^c^	1.34 ± 0.33 ^c^	0.46 ± 0.32 ^c^	0.18 ± 0.15 ^c^	0.64 ± 0.04 ^c^
TRIA2+Cd	2.17 ± 0.40 ^e^	0.61 ± 0.14 ^f^	2.78 ± 0.06 ^f^	0.91 ± 0.07 ^f^	0.41 ± 0.17 ^f^	1.32 ± 0.12 ^g^
TRIA3+Cd	1.87 ± 0.08 ^d^	0.49 ± 0.32 ^d^	2.36 ± 0.09	0.68 ± 0.03 ^d^	0.34 ± 0.37 ^e^	1.02 ± 0.23 ^e^

Values illustrate means ± SE of four replicates. Dissimilar letters on error bars showed significant differences among the treatments at *p* ≤ 0.05. C = control, Cd = 20 mg kg^−1^ Cd, TRIA1 = 10 µM L^−1^ TRIA, TRIA2 = 20 µM L^−1^ TRIA, TRIA3 = 30 µM L^−1^ TRIA.

**Table 3:** Impact of TRIA on plant pigments and Cd uptake, accumulation, and metal tolerance index in *V. radiata* under Cd stress.

Treatments	Chl *a* (mg g^−1^ FW)	Chl *b* (mg g^−1^ FW)	Total Chl (mg g^−1^ FW)	Carotenoids (mg g^−1^ FW)	Cd Uptake in Plant (mg/g)	AC Factor	MTI
C	0.74 ± 0.04 ^b^	1.43 ± 0.14 ^b^	2.15 ± 0.15 ^b^	0.08 ± 0.02 ^a^	-	-	-
Cd	0.45 ± 0.03 ^a^	0.81 ± 0.02 ^a^	1.27 ± 0.03 ^a^	0.03 ± 0.03 ^a^	0.84 ± 0.03 ^d^	42 ± 0.40 ^d^	55.56 ± 1.56 ^a^
TRIA 1	1.30 ± 0.03 ^c^	1.92 ± 0.06 ^cd^	3.21 ± 0.04 ^d^	0.13 ± 0.02 ^a^	-	-	-
TRIA 2	1.82 ± 0.05 ^e^	3.10 ± 0.06 ^f^	4.92 ± 0.02 ^g^	0.17 ± 0.02 ^a^	-	-	-
TRIA 3	1.42 ± 0.03 ^d^	2.68 ± 0.13 ^e^	4.11 ± 0.13 ^f^	0.12 ± 0.02 ^a^	-	-	-
TRIA 1+Cd	1.08 ± 0.05 ^c^	1.72 ± 0.03 ^c^	2.79 ± 0.04 ^c^	0.10 ± 0.03 ^a^	0.67 ± 0.02	33.5 ± 0.57 ^c^	177.78 ± 1.14 ^b^
TRIA 2+Cd	1.38 ± 0.07 ^d^	2.52 ± 0.03d ^e^	3.90 ± 0.04 ^e^	0.13 ± 0.02 ^a^	0.28 ± 0.01 ^a^	14 ± 0.60 ^a^	366.67 ± 0.90 ^d^
TRIA 3+Cd	1.21 ± 0.01 ^c^	2.19 ± 0.01 ^d^	3.40 ± 0.01 ^d^	0.11 ± 0.01 ^a^	0.45 ± 0.02 ^b^	22.5 ± 0.27 ^b^	283.33 ± 0.07 ^c^

Values illustrate means ± SE of four replicates. Dissimilar letters on error bars showed significant differences among the treatments at *p* ≤ 0.05. C = control, Cd = 20 mg kg^−1^ Cd, TRIA1 = 10 µM L^−1^ TRIA, TRIA2 = 20 µM L^−1^ TRIA, TRIA3 = 30 µM L^−1^ TRIA.

**Table 4:** Impact of TRIA on mineral content (mg g ^−1^) of *V. radiata* under Cd stress.

Treatments	Mg^+2^ (mg g^−1^)	Zn^+2^ (mg g^−1^)	K^+^ (mg g^−1^)	Na^+^ (mg g^−1^)
C	0.34 ± 0.60 ^b^	0.34 ±0.20 ^b^	17.82 ±1.20 ^b^	1.81 ± 0.04 ^b^
Cd	0.17 ± 0.27 ^a^	0.18 ± 0.40 ^a^	11.43 ± 0.09 ^a^	0.94 ± 0.08 ^a^
TRIA1	0.48 ± 0.60 ^c^	0.44 ± 0.05 ^c^	20.04 ± 0.43 ^d^	2.68 ± 0.16 ^c^
TRIA2	0.84 ± 0.64 ^g^	0.55 ± 0.08 ^d^	23.75 ± 0.09 ^f^	3.64 ± 0.09 ^d^
TRIA3	0.68 ± 0.16 ^e^	0.54 ± 0.26 ^d^	22.57 ± 0.05 ^e^	3.18 ± 0.07 ^d^
TRIA1 + Cd	0.38 ± 0.09 ^b^	0.42 ± 0.31 ^c^	18.78 ± 0.44 ^c^	2.26 ± 0.03 ^c^
TRIA2 + Cd	0.72 ± 0.40 ^f^	0.5 ± 0.07 ^cd^	22.49 ± 0.20 ^e^	3.05 ± 0.05 ^d^
TRIA3 + Cd	0.56 ± 0.04 ^d^	0.47 ± 0.05 ^c^	20.36 ± 0.15 ^d^	2.72 ± 0.03 ^c^

Values illustrate means ± SE of four replicates. Dissimilar letters on error bars showed significant differences among the treatments at *p* ≤ 0.05. C = control, Cd = 20 mg kg^−1^ Cd, TRIA1 = 10 µM L^−1^ TRIA, TRIA2 = 20 µM L^−1^ TRIA, TRIA3 = 30 µM L^−1^ TRIA.

## Data Availability

All the relevant required datasets can be obtained from the corresponding author on special request.
